# Cyclone exposure and mortality risk of children under 5 years old: An observational study in 34 low- and middle-income countries

**DOI:** 10.1371/journal.pmed.1004735

**Published:** 2025-09-25

**Authors:** Yichen Guo, Yixiang Zhu, Cheng He, Ya Gao, Lu Zhou, Jovine Bachwenkizi, Haidong Kan, Renjie Chen

**Affiliations:** 1 School of Public Health, Key Lab of Public Health Safety of the Ministry of Education, NHC Key Lab of Health Technology Assessment, Fudan University, Shanghai, China; 2 Department of Global Health and Population, Harvard T.H. Chan School of Public Health, Boston, Massachusetts, United States of America; 3 Department of Environmental and Occupational Health, Muhimbili University of Health and Allied Sciences, Dar es Salaam, Tanzania; 4 IRDR ICoE on Risk Interconnectivity and Governance on Weather/Climate Extremes Impact and Public Health, Fudan University, Shanghai, China; The Hospital for Sick Children, CANADA

## Abstract

**Background:**

Climate change has exacerbated the frequency, intensity, and impacts of extreme weather events (EWEs), such as tropical cyclones. However, the increasing impact of tropical cyclones on child mortality, especially in low- and middle-income countries (LMICs), remains understudied.

**Methods and findings:**

Utilizing individual-level data from the Demographic and Health Surveys, we conducted a sibling-matched case-control study to assess the impact of cyclone exposure over the past 3 months on under-five mortality. The study included 100,798 under-five deaths and 247,445 controls across 34 LMICs from 1993 to 2021. After adjusting for key meteorological and temporal confounders and maternal age, significant positive associations were observed between under-five deaths and cyclone exposure over the past 3 months before death (odds ratio [OR]: 1.038, 95% confidence interval [CI]: 1.002, 1.075; *p* = 0.041). Specifically, the strongest effects were observed in the first month before death (OR: 1.101, 95% CI: 1.039, 1.166; *p* < 0.001), diminishing in the second and third months before death. We estimated that cyclone exposure 0–30 days before death may have caused 0.85 million under-five deaths (95% CI: 0.35 million, 1.32 million) in countries exposed to cyclones from 2000 to 2020. As an observational study, its use of self-reported data and dichotomous exposure assessment may introduce recall bias and exposure misclassification, limiting accuracy and representativeness.

**Conclusions:**

This global analysis demonstrates the substantial under-five mortality risk from cyclones, emphasizing the importance of targeted strategies to enhance community resilience against the growing threat of EWEs on children, such as improving access to water sources and sanitation facilities.

## Author summary

**Why was this study done?:**

- Tropical cyclones are becoming more frequent and severe with climate change, raising concerns about their effects on young children in resource-limited countries.
- Existing studies often focus on single cyclone events or high-income regions, leaving a gap in understanding how cyclones impact child health across multiple low- and middle-income regions.

**What did the researchers do and find?:**

- We conducted a sibling-matched case-control analysis to examine the effect of tropical cyclones on children’s health in low- and middle-income countries (LMICs).
- This study includes 100,798 under-five deaths and 247,445 surviving siblings across 34 LMICs between 1993 and 2021.
- We observed increased under-five mortality risks associated with exposure to tropical cyclones in the month before death, estimating roughly 850,000 cyclone-related child deaths over two decades in the study area.

**What do these findings mean?:**

- Tropical cyclones pose a significant threat to child survival in vulnerable regions, underscoring the need for disaster preparedness that prioritizes water, sanitation, and health services.
- Strengthening community resilience through improved infrastructure and emergency planning, alongside ensuring equitable access to safe water and sanitation, could help protect children from cyclone impacts by reducing mortality risks linked to these events.
- As an observational study, its use of self-reported data and dichotomous exposure assessment may introduce recall bias and exposure misclassification, limiting accuracy and representativeness.

## Abstract 3

In a sibling-matched case-control study, Yichen Guo and colleagues assessed associations between cyclone exposure and mortality risk in children under five years old, in 34 low- and middle-income countries.

## Introduction

Tropical cyclones, including typhoons and hurricanes, are among the most prevalent and destructive natural disasters, posing significant threats to human health and life [1–3]. According to the American Meteorological Society (AMS) [4], and World Meteorology Organization (WMO) [5], based on maximum sustained wind speed and formation region, tropical cyclones are further classified as tropical depressions (<34 knots), tropical storms (34–63 knots), and for winds ≥64 knots, hurricanes in the Atlantic Ocean and northeastern Pacific Ocean, typhoons in the northwestern Pacific Ocean, or cyclones in the South Pacific and Indian Ocean. As estimated by WMO, over the past 50 years, tropical cyclones have caused at least 1,945 disasters worldwide, resulting in 77,000 direct deaths and USD 1.4 trillion in economic losses [6]. These extreme weather events (EWEs) are becoming increasingly frequent due to climate change and particularly devastating in low- and middle-income countries (LMICs) with limited resources and infrastructure for disaster response and mitigation [7–9]. Regions such as East Africa, South and Southeast Asia, and small island nations are projected to face increased tropical cyclones and extreme rainfalls in the future [10]. Consequently, the impact of tropical cyclones on human health is more severe in LMICs compared to developed countries. Studies have linked tropical cyclones to various health outcomes, including all-cause mortality [2,3,11], infectious diseases [12,13], and adverse peripartum outcomes [14,15], highlighting the substantial burden imposed by cyclones. However, existing research mostly originates from developed countries such as United States, leaving a significant gap in understanding the health impacts of cyclone exposure in LMICs [16,17]. A limited number of studies from LMICs have typically focused on individual high-intensity cyclone events and their impact on specific child health outcomes, resulting in limited representativeness and comparability across countries and regions [18–21].

There is also a notable lack of research focusing on the impact of EWEs on vulnerable populations, particularly young children, who may be disproportionately affected by these disasters. Children are especially vulnerable to tropical cyclones due to their physical, physiological, and social susceptibility, facing immediate threat from injuries caused by high winds, flying debris, and drowning from flooding [22,23]. Cyclones can also exacerbate various diseases during disaster events [24], further escalating the mortality risks among children [25,26]. For instance, exposure to Hurricane Sandy was found to increase long-term risks of symptom exacerbation among children with asthma [27], but no studies have yet examined the association between cyclone exposure and child mortality. In addition to vulnerability of children in LMICs to EWEs, varying socioeconomic characteristics of different subgroups within these countries may also modify cyclone-health associations. Cyclone damage can deteriorate water, sanitation, and hygiene (WASH) conditions and disrupt public service, further amplifying health risks [23,25]. The destruction of healthcare facilities and disruption of medical services after a cyclone can severely limit access to essential healthcare, impeding the timely treatment of injuries and illnesses [9,23,28,29]. Different socioeconomic characteristics can reflect resilience under EWEs, helping to identify vulnerable populations and implement precise interventions [30].

Given the distinctive vulnerabilities of children and the considerable health risks posed by tropical cyclones, this study seeks to address the existing research gap by investigating the associations between tropical cyclones and health outcomes in children from LMICs. Using a large-scale and multi-country database of children’s health in LMICs and a sibling-matched case-control design, we aimed to quantify the effects of cyclone exposure on deaths among children under 5 years old and assess the attributable burden associated with cyclone exposure. We also aimed to identify potential effect modifiers which may impact on the cyclone-child mortality associations based on possible mechanisms.

## Method and materials

### Study population

We collected individual-level health data of children in LMICs from the Demographic and Health Surveys (DHS) program, a global survey conducted in over 90 developing countries, covering maternal and children health, household conditions, and nutrition issues [31]. This survey adopted a 2-stage cluster sampling strategy [32]. First, urban and rural areas were stratified for each country included in the survey. Within each stratum, clusters were selected independently with probability proportional to their sizes. Subsequently, a complete list of households was created for each selected cluster, and households were chosen using equal probability sampling. In each selected household, trained fieldwork staff administered questionnaires to identify eligible interviewees and conduct further interviews.

To identify eligible deaths among children under 5 years old, we applied the following exclusion criteria. (1) Households lacking key information such as accurate geographic coordinates, interview date, and child’s age. (2) Households with only one child. (3) Records with abnormal information or interview dates earlier than 1985. (4) Countries that were not matched with any cyclones in given exposure time periods. (5) Survey records in which the number of interviewed households was fewer than 100.

We determined whether a child survived their first 5 years of life, and the length of survival time based on data on birth date, survival status, and death age of each child from DHS. For surviving children in the same household as the deceased child, we calculated the date when the control child reached the age of the deceased child based on the date of birth and the survival time of the deceased child, facilitating subsequent estimation of cyclone exposure. Specifically, for each deceased child, we matched all surviving siblings individually as controls at the age of the deceased child’s death to control for household-specific factors and age-related mortality risks. A detailed description of this matching process, including a schematic illustration (S1 Fig), is provided in the Supporting information .

We collected country- and age-specific mortality data from 2000 to 2020 from the United Nations Children’s Fund (UNICEF) Data Warehouse (https://data.unicef.org/dv_index/). Historical gridded age-specific population data were obtained from WorldPop (https://hub.worldpop.org/), with a spatial resolution of 1 km × 1 km from 2000 to 2020, which we further aggregated into a 10 km × 10 km grid. National-level GDP data and hospital beds per 1,000 population were acquired from the World Bank (https://data.worldbank.org/).

### Exposure data

We collected all available cyclone records from the Joint Typhoon Warning Center (JTWC, https://www.metoc.navy.mil/jtwc/jtwc.html) from 1985 to 2021. The JTWC provides track data for each cyclone worldwide since 1959, which includes information such as the cyclone’s center position, maximum wind speed, minimum pressure, and intensity level every 6 h along its track. This data serves as a crucial source for re-analyzing tropical cyclones and conducting environmental research.

We collected all available cyclone records from 1985 to 2021 and estimated corresponding buffer zones around the cyclone center positions using the R34 (34 kt or 63 km/h) cyclone radius, which is commonly used in environmental epidemiology research to determine cyclone exposure status [33]. These buffer zones were then matched with DHS clusters for each household prior to the death of each case child (or the control date for controls) to determine cyclone exposure. Exposure periods were classified into four categories: 0–30 days prior to the death/control date (lag 0 month), 31–60 days prior (lag 1 month), 61–90 days prior (lag 2 month), and 0–90 days prior (lag 0–2 months).

We also acquired temperature and precipitation data from ERA5, a global reanalysis dataset with a spatial resolution of 0.25° × 0.25° and up to 1-h temporal frequency [34]. Monthly mean temperature and cumulative precipitation were aggregated and matched to each household for different exposure periods.

### Statistical analyses

#### Associations between cyclone exposure and deaths among children.

This study adopted a sibling-matched case-control study design to examine the association between cyclone exposure and deaths among children under 5 years old [35,36]. By including deceased children as cases and their living siblings from the same mother as controls, this design effectively controls for time-invariant household-level confounders. This design has been widely applied in environmental epidemiological studies to investigate relationships between environmental exposures and health outcomes [35,36].

We utilized a conditional logistic regression model to quantify the association between cyclone exposure in different lag periods and under-five deaths. We accounted for meteorological confounders by incorporating moving averages of monthly temperature and cumulative precipitation during the exposure period, using natural cubic spline functions with six degrees of freedom. Additionally, to control for potential time trends, we adjusted for the year and month of child death (or the year and month when the control child reached the age of the deceased child) and mother’s age at the time of death/control. We also conducted an analysis using infant death (death of a child less than 1-year old) as a secondary outcome to evaluate whether children under 1-year old may be at higher risk. The cyclone-health association was estimated by calculating the odds ratios (ORs) and corresponding 95% confidence interval [CI] using following equations [37]:


$$OR=e^{\beta }$$ (1)

$$Lower\;95\%\;confidence\;Interval\;of\;OR=e^{\beta -1.96\times se}$$ (2)

$$Higher\;95\%\;confidence\;Interval\;of\;OR=e^{\beta +1.96\times se}$$ (3)

where *β* is the coefficient derived from the regression model, and se is the standard error of the regression coefficient.

Stratified analyses were conducted to explore potential modification effects of social-economic variables, such as household water sources and toilet types based on previous literatures [9,22,23,25,26]. We extracted the following sociodemographic variables from the DHS database for stratified analyses: household residence area (rural and urban), children’s gender (male and female), child birth order (firstborn and nonfirstborn), maternal highest education (primary or no education, secondary education, high school and above), household drinking water source (piped or bottled water, well water, natural water, others), household sanitation facility (flush toilet, pit toilet, no toilet, others) and housing construction materials. Housing construction materials were coded as “finished materials” if at least two of the three structural components (floor, ceiling, and walls) were primarily built with finished materials. In addition, using World Bank data and a median split, we defined regional GDP per capita and regional medical resource level as lower than average and higher than average.

We applied a *Z*-test to compare effect estimates between any two subgroups as [37,38]:


$$Z=\frac{\beta_{1}-\beta_{2}}{\sqrt{{SE}_{1}^{2}+{SE}_{2}^{2}}}$$ (4)

where $\beta_{1}$ and $\beta_{2}$ are the coefficient estimates for cyclone-related deaths in two subgroups from stratified analyses, and SE_1_ and SE_2_ are their standard errors, respectively. We then derived a two‐sided *p* value from the standard normal distribution to determine whether the intergroup difference was statistically significant.

To further explore possible pathways through which tropical cyclones affect child mortality, we conducted supplementary analyses using data on children’s diarrhea symptoms from the DHS program. Employing the same cyclone exposure assessment method and sibling-matched design, we examined the association between cyclone exposure and the prevalence of diarrhea symptoms in children under 5 years old. Using the same covariate adjustments and model specifications as the primary analysis, we performed stratified analyses to assess potential effect modification by household water sources and sanitation facilities on the relationship between cyclone exposure and diarrhea.

To verify the robustness of our main model, we conducted several sensitivity analyses. First, we adjusted the degrees of freedom of temperature and precipitation from 6 to 3. Second, we included the covariate of child’s gender as an additional covariate in the main model. Third, we removed the covariates of time trends (the year and month of child death). Finally, we adopted a “leave-one-out cross-validation” approach [39–41] to identify potential influential data and examine the robustness of our model. In this method, data from each country was excluded from the dataset one at a time, and the model was reevaluated to assess the impact of excluded data on the overall estimations.

#### Estimation of deaths among children attributable to cyclone exposure.

We estimated excess under-five deaths attributable to cyclone exposure based on previous effects estimates and following method [42]:


$$ED_{g,y}=\frac{RR-1}{RR}\times P_{g,y}\times M_{g,y}\times C_{g,y}$$ (5)

where $\text{E}{\text{D}}_{g,y}\;$ represents excess deaths of grid g in year y. RR is the relative risk of mortality under 5 years old in children exposed to cyclones in the past month compared to those without cyclone exposure. Given the large sample size of our study and the relatively low under-five mortality rates observed across the study regions, we followed standard practice in environmental epidemiology by treating the model-derived ORs as approximations of relative risks (RRs) in all subsequent analyses [43,44]. Similarly, we used the upper and lower bounds of the OR CIs to estimate the corresponding range of attributable fractions. $P_{g,y}\;$ represents the population of children under 5 years old of grid g in year y, while $M_{g,y}\;$ represents the mortality rate for children under 5 years old of grid g in year y. The grid-specific mortality rate was approximated using the national mortality rate for the country to which the grid belongs. Using $P_{g,y}$ and $M_{g,y}$, we calculated the number of deaths among children under 5 years old in each grid from 2000 to 2020. $C_{g,y}\;$represents the numbers of cyclone exposure of grid g in year y.

The publicly available population data used in this study have been reviewed and approved by the Institutional Review Board of ICF Incorporated. All statistical analyses were conducted in R (version 3.6.3) with the “*survival*” package. All analyses were two-sided with an α of 0.05 and *p* value < 0.05 was considered statistically significant. This study is reported as per the Strengthening the Reporting of Observational Studies in Epidemiology (STROBE) guideline (S1 STROBE Checklist).

## Results

### Descriptive results

**Fig 1:**
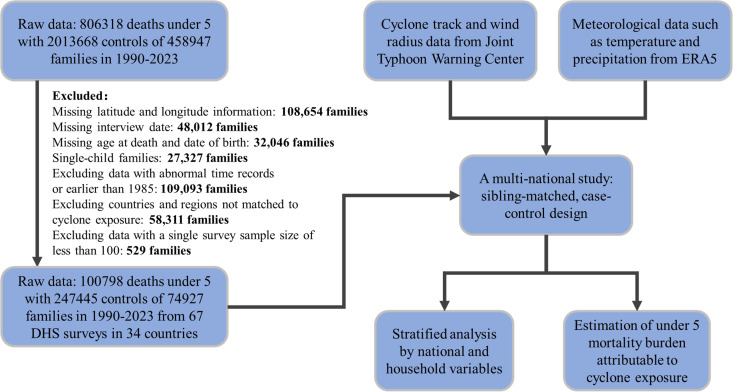
Diagram of data preparations.

**Fig 2:**
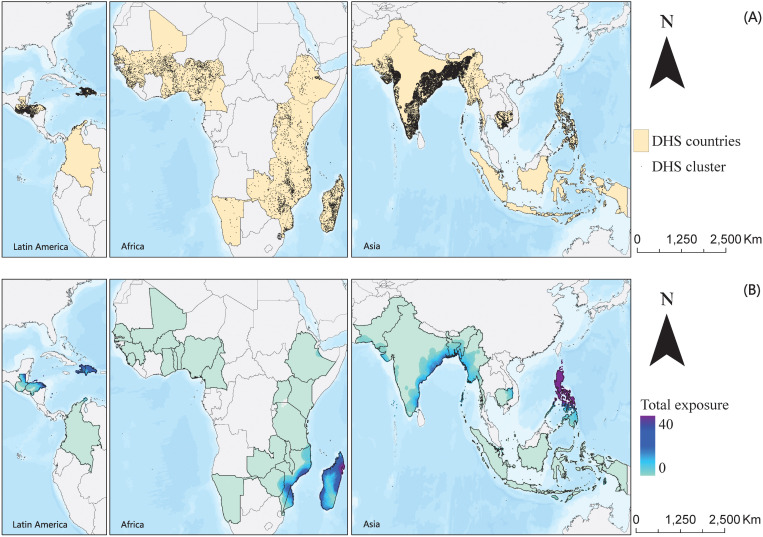
Spatial distribution of included DHS clusters (A) and 10 × 10 km raster level exposure of cyclones (B) in 2000–2020. Note: This map was reproduced from https://www.naturalearthdata.com/.

This study included 348,243 children under 5 years old, comprising 100,798 deaths (mean [SD] age of death, 23.0 [6.0] months) and 247,445 controls from 67 DHS surveys conducted in 34 LMICs across Africa, Asia, and Latin America from 1993 to 2021 (Fig 1). The geographical distribution of clusters is shown in Fig 2A. Each case was matched with an average of 2.5 controls. Approximately 68% of the cases were from Asia, 79% of deaths occurred in children under 1 year old, 87% lived in rural areas, and only 30% had secure access to drinking water or flush toilet (Table 1 and S1 Table).

**Table 1:** Summary characteristics of the population from case group and control group.

Variables	Cases^a^	Controls
	(*N* = 100,798)	(*N* = 247,445)
Death age (months old)		
0–11	80,115 (79.48%)	NA
12–23	10,891 (10.8%)	NA
24–35	4,773 (4.74%)	NA
36–47	2,636 (2.62%)	NA
48–59	2,383 (2.36%)	NA
Region		
Asia	68,711 (68.17%)	158,993 (64.25%)
Africa	19,401 (19.25%)	50,611 (20.45%)
Latin America	12,686 (12.59%)	37,841 (15.29%)
Residence area		
Urban	22,059 (21.88%)	52,003 (21.02%)
Rural	78,739 (78.12%)	195,442 (78.98%)
Children’s gender		
Male	55,612 (55.17%)	124,149 (50.17%)
Female	45,186 (44.83%)	123,296 (49.83%)
Birth order		
First child	35,242 (34.96%)	36,816 (14.88%)
Not first child	65,556 (65.04%)	210,629 (85.12%)
Mother’s highest education		
Primary or no education	77,594 (76.98%)	200,336 (80.96%)
Secondary education	21,151 (20.98%)	43,474 (17.57%)
High school or above	2,052 (2.04%)	3,629 (1.47%)
Regional GDP per capita		
Lower than average	27,629 (27.41%)	74,091 (29.94%)
Higher than average	73,169 (72.59%)	173,354 (70.06%)
Regional medical resource		
Lower than average	33,259 (33.00%)	89,744 (36.27%)
Higher than average	67,539 (67.00%)	157,701 (63.73%)
Water sources		
Piped or bottled water	32,863 (32.6%)	78,629 (31.78%)
Well water	45,750 (45.39%)	109,916 (44.42%)
Natural water	12,809 (12.71%)	35,191 (14.22%)
Others	9,376 (9.3%)	23,709 (9.58%)
Toilet types		
Flush toilet	28,802 (28.57%)	66,760 (26.98%)
Pit toilet	32,148 (31.89%)	84,098 (33.99%)
No toilet	35,141 (34.86%)	84,445 (34.13%)
Others	4,707 (4.67%)	12,142 (4.91%)
Household materials		
Unfinished	55,692 (55.25%)	139,594 (56.41%)
Finished	45,106 (44.75%)	107,851 (43.59%)
Temperature^b^ (°C)	17.24 ± 10.48	18.30 ± 10.45
Precipitation^b^ (mm)	201.79 ± 200.19	190.26 ± 196.49

^a^Missing data for some stratification variables may result in figures not adding up to group totals or percentages not adding up to 100%.

^b^The average temperature or cumulative precipitation in the month before death for cases or in the previous month at the same age for matched controls. Values are displayed as mean ± standard error.

From 2000 to 2020, we estimated ~98.36 million deaths among children under 5 years old in the 34 DHS countries. During this period, we identified 358 cyclone events affecting 18 of these countries, primarily in Latin America (e.g., Haiti and the Dominican Republic), Southeast Asia (e.g., Philippines and Myanmar), the East Africa (e.g., Mozambique and Madagascar), and coastal area of South Asia (e.g., Bangladesh and India) (Fig 2B).

### Regression results

**Fig 3:**
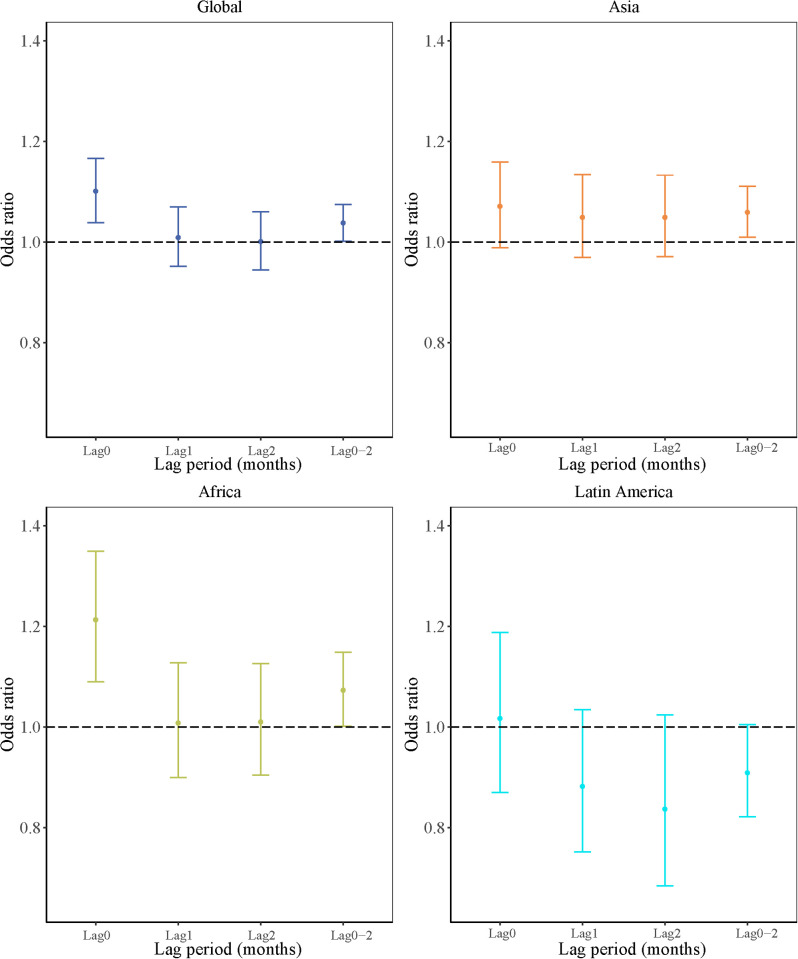
Odds ratios (and 95% confidence intervals) of death risks in children under 5 years old associated with cyclone exposure globally and regionally in different lag periods before death. The dots represent the mean effect estimates, and the lines are their 95% confidence intervals.

We observed significant positive associations between cyclone exposure within the past 3 months prior to death and deaths among children under 5 years old globally, with an OR of 1.038 (95% CI: 1.002, 1.075; *p* = 0.042) (Fig 3 and S2 Table). Specifically, the strongest effects were observed in the first month before death (0–30 days prior to death; OR: 1.101, 95% CI: 1.039, 1.166; *p* < 0.001), diminishing in the second month before death (31–60 days) and third month before death (61–90 days). Similar trends were observed in Africa, with an OR of 1.073 (95% CI: 1.002, 1.149; *p* = 0.045) for the past 3 months prior to death and OR for the first month prior to death of 1.213 (95% CI: 1.090, 1.349; *p* < 0.001). However, the effect of cyclone exposure was significant only within the past 3 months preceding death in Asia (OR: 1.059, 95% CI: 1.010, 1.111; *p* = 0.017). No evidence supported such an association in Latin America.

**Fig 4:**
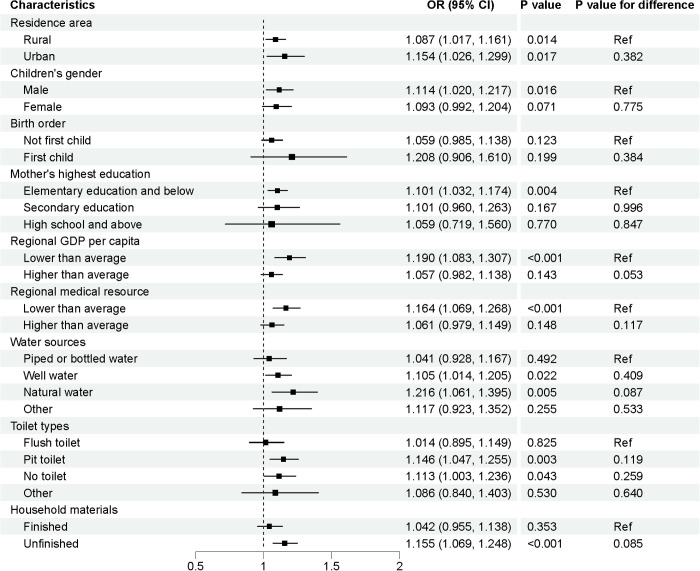
Odds ratios (and 95% confidence intervals) of death risks in children under 5 years old associated with cyclone exposure in the first month before death globally in various subgroups. “Lower than average” in the 5th category represents that the family interviewed lived in a country with lower than average GDP per capita of all included countries; “Lower than average” in the 6th category represents that the number of hospital bed per 1,000 population in a country was lower than the average of all included countries; “Finished” in the 9th category represents that the family interviewed lived in a house with at least two parts using finished rather than natural or rudimentary material among roof, floor or wall. The dots are odds ratios of under-five deaths associated with cyclone exposure, and the lines are their 95% confidence intervals.

As shown in Fig 4 and S3 Table, the impact of cyclone exposure in the month preceding death on child mortality appeared more pronounced among children living in urban areas or first-borns, although these differences were not statistically significant. Significant cyclone-child mortality associations were only identified in male children, children of mothers with primary or no education, children living in regions with below-average GDP per capita and below-average regional medical resource, and those from households relying on well or natural water, using pit toilet or no toilet, and primarily utilizing rudimentary housing material. Stratified effect estimates for cyclone exposures across different lags are detailed in S4 Table.

In sensitivity analyses, effect estimates remained robust in models after adjusting for degrees of freedom of temperature and precipitation, covariates of children sex and time trends (S5 Table). The “leave-one-out cross-validation” analysis further confirmed the stability of our primary model when data from each DHS country were excluded one by one (S6 Table).

Using infant mortality as a secondary outcome, we observed similar effect estimates to the main model (OR: 1.109, 95% CI: 1.036, 1.186; *p* = 0.003) in the first month before death (S7 Table). Supplementary analyses indicated a significant positive association between cyclone exposure within the past 3 months before disease and diarrhea symptoms in children under 5 years old in LMICs (OR: 1.224, 95% CI: 1.144, 1.309; *p* < 0.001) (S8 Table). Similarly, higher cyclone-diarrhea effect estimates were found in households using well or natural water compared to piped or bottled water, and in households with pit or no toilets compared to those with flush toilets. However, no statistically significant differences were observed between strata.

#### Attributable deaths among children under 5 years old.

**Fig 5:**
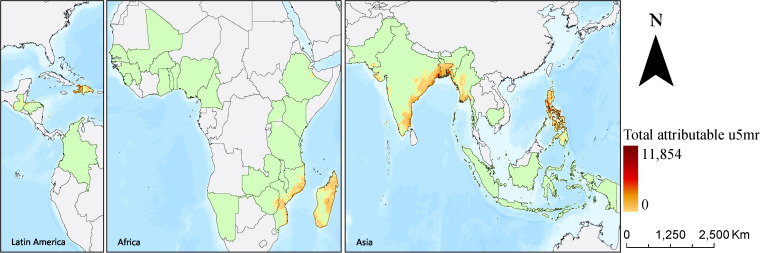
Spatial distribution of 10 × 10 km raster level deaths in children under 5 years old attributable to cyclone exposure in 2000–2020. Note: This map was reproduced from https://www.naturalearthdata.com/.

We estimate that a single cyclone exposure could account for ~9.17% (95% CI: 3.75%, 14.23%; *p* < 0.001) of deaths among children under 5 years old in the month preceding death globally, with a higher proportion in Africa at 17.56% (95% CI: 8.26%, 25.87%; *p* < 0.001). According to our estimates, from 2000 to 2020 across the 34 DHS countries included in the analysis, the average annual under-five population was ~307.84 million, and the average annual number of under-five deaths was ~4.68 million, matching the figures reported by UNICEF for these countries (S9 Table). Over the same period, we estimate that 0.85 million deaths (95% CI: 0.35, 1.32 million; *p* < 0.05) in children under 5 years old, accounting for 8.67% (95%CI: 3.55%, 13.46%; *p* < 0.05) total under-five deaths, could be attributable to cyclone exposure in the first month before death across included DHS countries (S10 Table). As illustrated in Fig 5, regions with the highest cumulative number of attributable deaths between 2000 and 2020 are primarily coastal areas in South and Southeast Asia, East Africa, and island nations in the Caribbean. Among the three regions, Asia had the highest estimated number of attributable child deaths at 3.1 million (95% CI: 1.3, 4.8 million; *p* < 0.05). Furthermore, excess child mortality rates attributable to cyclones were higher in Latin America and Africa, with 15.2 and 2.46 deaths per 10,000 children, respectively, compared to 3.9 deaths per 10,000 children in Asia.

## Discussion

This large-scale, multi-country epidemiological study assessed the significantly positive association (OR: 1.101, 95% CI: 1.039, 1.166; *p* < 0.001) between tropical cyclone exposure and deaths in children under 5 years old in LIMCs. We further provided novel evidence on the burden of under-five mortality, estimating 0.85 million deaths attributable to cyclone exposure in DHS countries from 2000 to 2020. Insecure access to drinking water and inadequate sanitation facilities may amplify these effects, highlighting the urgent need for targeted WASH interventions to improve water and sanitation infrastructure in cyclone-prone areas to protect children’s health.

The increased risk of under-five mortality associated with cyclone exposure aligns with the health risks posed by EWEs for children in previous research. For instance, a study in Queensland, Australia, reported increased risks of preterm birth (OR: 1.28, 95% CI: 1.11, 1.49; *p* < 0.05) and low birth weight (OR: 1.62, 95% CI: 1.00, 2.40; *p* < 0.05) in areas affected by severe cyclones during early-to-mid pregnancy [45]. Another study found higher risks of compounded morbidity (OR: 1.43, 95% CI: 1.08, 1.92; *p* < 0.05) and mortality (OR: 43.2, 95% CI: 5.03, 370.6; *p* < 0.05) in newborns of women affected by Hurricane Harvey [14]. A study of the 1931 hurricane in Fiji suggested that the hurricane and subsequent flooding might have contributed to a mortality rate of up to 16.5/1,000 among children in the country’s floodplains [46]. However, previous findings were less conclusive regarding child mortality. One study claimed no significant difference in child mortality rates estimated from local newspaper reports before and after Hurricane Katrina [47]. Another study in the United Kingdom observed more deaths in the year preceding flood exposure compared to the year following it, attributing this to the potential impact of migration resulting from flooding [48]. These mixed findings may stem from differences in study design, sample size, and regional heterogeneity, hindering comparability and extrapolation. By utilizing the sibling-matched design and data from multiple LMICs globally, our study provides stronger and more generalizable evidence.

Cyclone exposure may increase child mortality risks through several pathways. First, children are particularly vulnerable to drowning and injuries during cyclones [22,23,49], due to their curiosity and weak perception of danger, as well as the absence of supervising adults during EWEs [50]. Second, cyclones can damage infrastructure and disrupt essential health services, limiting timely medical care. Multiple studies demonstrated the negative impact of EWEs, such as hurricanes and floods, on healthcare utilization [15,18,20,28,29,51,52], particularly in LMICs. A study based on county-level statistics in the United States indicates that highly destructive EWEs lead to increased unscheduled hospital visits, overburdening health service, and increasing mortality rates in the affected counties [52]. This hypothesis is consistent with findings in our stratified analysis, where we found that in regions with relatively scarce medical resources, cyclone exposure is still associated with an increased risk of under-five mortality in the area, whereas in regions with relatively adequate medical resources, this association is no longer significant. Third, cyclones and subsequent flooding can worsen living conditions, increasing the risk of malnutritional and infectious diseases [24,53]. Excessive rainfall or floods also pose significant risks for microbiological or fecal water contamination [25,54], leading to higher incidences of diseases such as diarrhea [12,25,26], a major cause of children mortality [22,55,56]. A study from Guangdong, China, found a significant increase in infectious diarrhea (hazard ratios [HR]: 1.95, 95% CI: 1.22, 3.12; *p* < 0.05) after tropical cyclones [12]. An African study likewise found that increased seasonal rainfall may amplify regional malaria transmission risk, possibly by providing stagnant water bodies necessary for the larval stages of Anopheles mosquitoes and boosting the population of adult mosquitoes capable of transmitting the Plasmodium parasite, thereby elevating the risk of malaria infection and mortality risk in children [57]. However, peaks in infectious diseases after heavy rainfall or flooding were only observed in developing countries, likely due to the lack of robust and adequate water supply infrastructures in developed countries [58,59]. Our supplementary analyses also support these hypotheses, revealing a significant association between cyclone exposure and diarrhea among children in the current month from a multi-country perspective, with insecure access to drinking water and inadequate sanitation facilities amplifying this association. However, as diarrhea data only included children who survived until the survey, the difference in sample population limited our ability to conduct mediation analyses.

We found significant cyclone-related mortality risks among male children and those whose mother had primary or no education. Evidence on gender differences in EWEs remains mixed. Some studies reported higher flood-related mortality rate among girls because of societal inequality [42,60], while other evidence suggested higher risks for boys due to their vulnerability to severe injuries and drowning during hurricanes [49]. Mothers with limited education may experience elevated stress during EWEs, impairing their ability to provide adequate care [23]. Furthermore, children in households lacking secure water sources, adequate sanitation facilities, and durable housing materials are at heightened risks. Excessive rainfall and flooding from cyclones can contaminate groundwater with untreated sewage and toxic waste, increasing the risk of gastrointestinal infections [25,54,61]. Additionally, children in areas with lower GDP per capita often lack adequate health resources, and cyclone-induced service disruption may exacerbate mortality risk [28,51,61].

To our knowledge, no studies have quantified the burden of under-five deaths attributable to tropical cyclones. Our findings estimated ~0.85 million under-five deaths attributable to cyclone exposure, with an excess actual under-five mortality rate of ~1.67 per 10,000 under-five children in the included DHS countries between 2000 and 2020. A study based on national survey data from Vietnam indicates that EWEs may have increased the national infant mortality rate by ~0.29 per 10,000 live births (95% CI: 0.11, 0.48; *p* < 0.05) between 1999 and 2018 [62]. In our study, we observed that in Cambodia, which shares significant geographical similarities with Vietnam, an excess actual under-five mortality rate of ~0.13/10,000 under-five children could be attributed to cyclone exposure between 2000 and 2020. With climate change increasing the frequency and severity of tropical cyclones, their impact on children mortality is expected to intensify [63,64]. Similar to the distribution of cyclone hotspots, the cyclone-related child mortality showed highest in island and coastal areas. Notably, even minor cyclone exposure in densely populated coastal regions may contribute to disproportionately high child deaths, highlighting the sensitivity of these regions to climate change-induced EWEs [65]. Most of the world’s megacities are located in coastal regions [66]. Recently, growth in land use and population has been significantly higher in coastal regions than inland regions, particularly in developing countries of East Asia, South Asia, and Africa [67]. Even under the most optimistic estimates, population in low-elevation coastal regions is projected to increase by 122% by 2060 compared to 2000 [68]. Growing population, aging infrastructure and encroachment of sea-level rise would put overpopulated coastal cities at higher risks from EWEs such as typhoons [69]. Our findings underscore a significant public health concern: children in households without secure WASH facilities face a markedly higher risk of cyclone-related illnesses and mortality. From a policy perspective, these findings advocate for prioritizing water and sanitation infrastructure in cyclone-prone coastal regions of LMICs as a critical component of disaster preparedness and response. Integrating these infrastructural improvements with broader public health initiatives can enhance community health and resilience, reducing the burden of disease and mortality associated with cyclones [69].

Despite the public health implications in this study, several limitations need to be acknowledged. First, reliance on self-reported data inevitably introduces recall bias. Specifically, inaccuracies in the exact dates of birth and death may affect our assessment of actual cyclone exposure. Second, the cluster-based sampling method could lead to exposure misclassification, as we only considered areas within a certain wind radius but did not consider secondary hazards like flooding. Additionally, dichotomous exposure assessment based on wind speed thresholds may also underestimate the stronger health hazards posed by higher wind speeds in the hurricane’s core area, highlighting the need for future research to adopt more refined exposure assessments. Third, the OR were found to be close to 1, indicating that the study results could still be attributed to a small number of unmeasured confounders, despite the efforts made in this study. Therefore, the interpretation of the results should be more cautious. Fourth, the inherent heterogeneity within countries and the exclusion of single-child families may limit the representativity and generalizability of our findings. Also, the results may not be generalizable to areas with different climatic characteristics, as most of the included LMICs are in monsoon climates. Finally, differences in sample sizes and exposure conditions between countries were not fully accounted for. Consequently, using a uniform global effect estimate for each region increases the uncertainty in our estimation of child deaths attributable to cyclone exposure.

This study, spanning 34 LMICs globally, provides valuable insights into the associations between tropical cyclone exposure over the past 3 months and increased mortality risks for children under 5 years old. It also reveals spatial heterogeneity in cyclone exposure and attributable child deaths in DHS countries between 2000 and 2020, with densely populated coastal areas being particularly vulnerable. Our findings also suggest potential mitigating role of adequate water and sanitation conditions in cyclone-prone areas, calling for targeted research and interventions.

## Supporting information

S1 Methods Detailed description of the matching process for sibling-matched case-control study design. (DOCX)

S1 Fig A schematic illustration of the matching process for a hypothetical family with five children. (DOCX)

S1 Table Summary characteristics of the population from groups with and without cyclone exposure 90 days before interview. (DOCX)

S2 Table Odds ratios (and 95% confidence intervals) of death risks in children under 5 years old associated with exposure of cyclone in the first month before death globally and regionally. (DOCX)

S3 Table Odds ratios (95% confidence intervals) of death risks in children under 5 years old associated with exposure of cyclone in the first month before death in models of stratification analyses. (DOCX)

S4 Table Odds ratios (95% confidence intervals) of death risks in children under 5 years old associated with exposure of cyclone in models of stratification analyses. (DOCX)

S5 Table Odds ratio (95% confidence intervals) of death risks in children under 5 years old associated with exposure of cyclone in the first month before death in models of sensitivity analyses. (DOCX)

S6 Table Odds ratio (95% confidence intervals) of death risks in children under 5 years old associated with exposure of cyclone in the first month before death after excluding every single country. (DOCX)

S7 Table Odds ratio (95% confidence intervals) of death risks in children under 1 years old associated with exposure of cyclone. (DOCX)

S8 Table Odds ratios (and 95% confidence intervals) of diarrhea risks in children under 5 years old associated with cyclone exposure, stratified by water sources and toilet types. (DOCX)

S9 Table Comparison of annual estimated population and deaths of children under-five in DHS countries (2000–2020) with UNICEF and World Bank database. (DOCX)

S10 Table Total deaths in children under 5 years old attributable to cyclone exposures in the first month before death in DHS countries exposed to cyclones from 2000 to 2020. (DOCX)

S1 STROBE Checklist This checklist is licensed under the Creative Commons Attribution 4.0 International License (CC BY 4.0; https://creativecommons.org/licenses/by/4.0/).
(DOCX)

## Abbreviations

AMS: American Meteorological Society
CI: confidence interval
DHS: Demographic and Health Surveys
EWE: extreme weather event
HR: hazard ratio
JTWC: Joint Typhoon Warning Center
LMICs: low- and middle-income countries
OR: odds ratio
RR: relative risk
STROBE: Strengthening the Reporting of Observational Studies in Epidemiology
WMO: World Meteorology Organization.
