# Supplementary material for: Cyclone exposure and mortality risk of children under 5 years old: An observational study in 34 low- and middle-income countries
Source: PLoS Med. 2025 Sep 25;22(9):e1004735. doi: 10.1371/journal.pmed.1004735 (PMC12463208; doi:10.1371/journal.pmed.1004735)
Supplement: S1 Methods — (DOCX) [file pmed.1004735.s001.docx]

**sMethod**

Detailed Description of the Matching Process for sibling-matched case-control study design

To investigate the relationship between cyclone exposure and child mortality, we utilized a sibling-matched case-control study design. This approach controls for stable, household-specific factors (e.g., socioeconomic status, parental care practices) and age-related mortality risks by comparing deceased children (cases) with their surviving siblings (controls) at the same age as the deceased child’s death. For each child who died before reaching the age of five, we identified all surviving siblings within the same household and matched them individually as controls at the age at which the deceased child passed away. This ensures that both cases and controls are evaluated at equivalent points in their lives, enabling a precise assessment of cyclone exposure and its impact on survival.

Consider a hypothetical family with five children, labeled A, B, C, D, and E, as an example. In this family, Child A died at age 1, Child B died at age 3, and Children C, D, and E survived beyond age five. The matching process for this family proceeds as follows:

Matching for Child A (Deceased at Age 1): At the time of Child A’s death (age 1), we assessed the survival status of all other siblings—Children B, C, D, and E—at age 1. Since Child B had not yet reached age 3 (and thus was still alive at age 1), and Children C, D, and E also survived to at least age 1, all four siblings (B, C, D, and E) were matched as controls for Child A. This matching is based on their status at age 1, calculated using their birth dates and Child A’s survival time (1 year).

Matching for Child B (Deceased at Age 3): At the time of Child B’s death (age 3), we assessed the survival status of the remaining siblings—Children C, D, and E—at age 3. By this point, Child A had already died at age 1 and thus could not be included as a control. However, Children C, D, and E, who all survived beyond age 3, were matched as controls for Child B. This matching is based on their status at age 3, calculated using their birth dates and Child B’s survival time (3 years).

This process is visually represented in Supplementary Figure 1, which illustrates the timelines of all five children. Child A’s timeline ends at age 1 (marked with a blue 'X'), and Child B’s timeline ends at age 3 (marked with an orange 'X'). The timelines for Children C, D, and E extend beyond age 5, indicating their survival past this threshold. Blue dashed lines connect Child A’s death at age 1 to the points on the timelines of Children B, C, D, and E at age 1, showing their use as controls for Child A. Similarly, orange dashed lines connect Child B’s death at age 3 to the points on the timelines of Children C, D, and E at age 3, showing their use as controls for Child B. This within-household matching strategy ensures that controls are drawn from the same family as the case, inherently adjusting for shared household characteristics, while also aligning the age of controls with the age at death of the case, thereby accounting for age-specific mortality risks.
