# Supplementary material for: Cyclone exposure and mortality risk of children under 5 years old: An observational study in 34 low- and middle-income countries
Source: PLoS Med. 2025 Sep 25;22(9):e1004735. doi: 10.1371/journal.pmed.1004735 (PMC12463208; doi:10.1371/journal.pmed.1004735)
Supplement: S1 Fig — (DOCX) [file pmed.1004735.s002.docx]

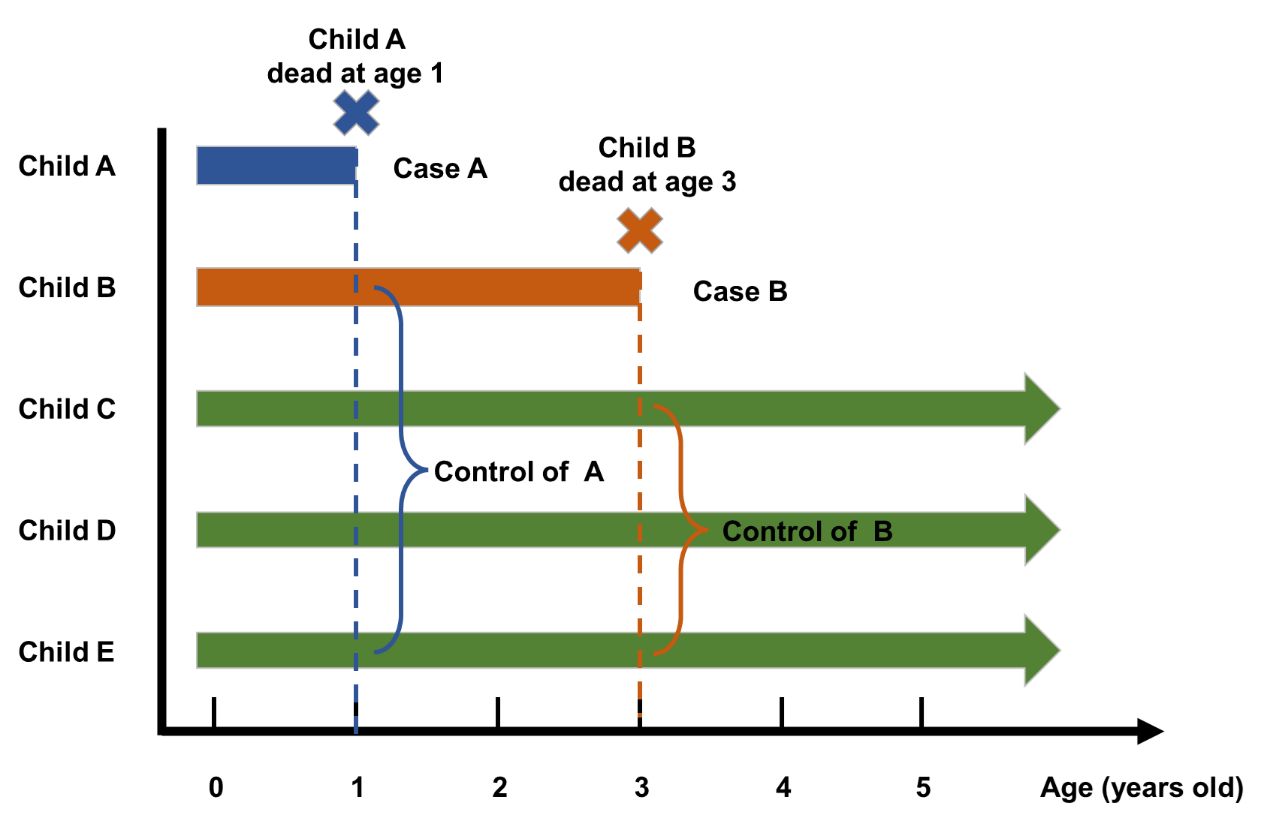


**S1 Fig. A schematic illustration of the matching process for a hypothetical family with five children.** Child A (blue bar) died at age 1, and Child B (orange bar) died at age 3. Children C, D, and E (green bars) survived beyond age 5. Blue dashed lines and arrows indicate the matching of siblings B, C, D, and E as controls for Child A at age 1. Orange dashed lines and arrows indicate the matching of siblings C, D, and E as controls for Child B at age 3. The x-axis represents age in years, ranging from 0 to 5.
