# Supplementary material for: Cyclone exposure and mortality risk of children under 5 years old: An observational study in 34 low- and middle-income countries
Source: PLoS Med. 2025 Sep 25;22(9):e1004735. doi: 10.1371/journal.pmed.1004735 (PMC12463208; doi:10.1371/journal.pmed.1004735)
Supplement: S1 Table — (DOCX) [file pmed.1004735.s003.docx]

**S1 Table. Summary characteristics of the population from groups with and without cyclone exposure 90 days before interview.**

| Variables | Exposed group ^a^ | Unexposed group |
| --- | --- | --- |
|  | (N=4,548) | (N=96,250) |
| Death age (months old) |  |  |
| 0–11 | 3,505 (77.07%) | 82,901 (86.13%) |
| 12–23 | 604 (13.28%) | 7,497 (7.79%) |
| 24–35 | 199 (4.38%) | 2,955 (3.07%) |
| 36–47 | 124 (2.73%) | 1,587 (1.65%) |
| 48–59 | 116 (2.55%) | 1,370 (1.42%) |
| Region |  |  |
| Asia | 2,695 (59.26%) | 66,016 (68.59%) |
| Africa | 1,178 (25.9%) | 18,223 (18.93%) |
| Latin America | 675 (14.84%) | 12,011 (12.48%) |
| Residence area |  |  |
| Urban | 1,075 (23.64%) | 21,045 (21.86%) |
| Rural | 3,473 (76.36%) | 75,205 (78.14%) |
| Children’s gender |  |  |
| Male | 2,507 (55.12%) | 53,105 (55.17%) |
| Female | 2,041 (44.88%) | 43,415 (45.11%) |
| Birth order |  |  |
| First child | 1,551 (34.1%) | 33,691 (35%) |
| Not first child | 2,997 (65.9%) | 62,559 (65%) |
| Mother’s highest education |  |  |
| Primary or no education | 3,508 (77.13%) | 74,086 (76.97%) |
| Secondary education | 913 (20.07%) | 20,238 (21.03%) |
| High school or above | 126 (2.77%) | 1,926 (2%) |
| Regional GDP per capita |  |  |
| Lower than average | 1,675 (36.83%) | 25,954 (26.97%) |
| Higher than average | 2,873 (63.17%) | 70,296 (73.03%) |
| Regional medical resource |  |  |
| Lower than average | 2,077 (45.67%) | 31,182 (32.40%) |
| Higher than average | 2,471 (54.33%) | 65,068 (67.60%) |
| Water sources |  |  |
| Piped or bottled water | 1,272 (27.97%) | 31,591 (32.82%) |
| Well water | 2,129 (46.81%) | 43,621 (45.32%) |
| Natural water | 753 (16.56%) | 12,056 (12.53%) |
| Others | 394 (8.66%) | 7,386 (7.67%) |
| Toilet types |  |  |
| Flush toilet | 1,100 (24.19%) | 27,702 (28.78%) |
| Pit toilet | 1,816 (39.93%) | 30,332 (31.51%) |
| No toilet | 1,395 (30.67%) | 33,746 (35.06%) |
| Others | 237 (5.21%) | 4,470 (4.64%) |
| Household materials |  |  |
| Unfinished | 2,496 (54.88%) | 53,197 (55.27%) |
| Finished | 2,052 (45.12%) | 43,054 (44.73%) |
| Temperature ^b^ (℃) | 20.68±9.24 | 16.94±10.24 |
| Precipitation ^b^ (mm) | 1,588.82±3,159.92 | 1,593.98±3,305.36 |

Notes:

^a^ Missing data for some stratification variables may result in figures not adding up to group totals or percentages not adding up to 100%.

^b^ The average temperature or cumulative precipitation in the month before death for cases or in the previous month at the same age for matched controls. Values are displayed as mean ± standard error.
