# Supplementary material for: Cyclone exposure and mortality risk of children under 5 years old: An observational study in 34 low- and middle-income countries
Source: PLoS Med. 2025 Sep 25;22(9):e1004735. doi: 10.1371/journal.pmed.1004735 (PMC12463208; doi:10.1371/journal.pmed.1004735)
Supplement: S2 Table — (DOCX) [file pmed.1004735.s004.docx]

**S2 Table. Odds ratios (and 95% confidence intervals) of death risks in children under 5 years old associated with exposure of cyclone in the first month before death globally and regionally.**

| Region | Lag (month) | Odds ratio (95% CI) | P value |
| --- | --- | --- | --- |
| Global | Lag 0 | 1.101 (1.039, 1.166) | <0.001 |
| Global | Lag 1 | 1.009 (0.952, 1.070) | 0.754 |
| Global | Lag 2 | 1.001 (0.945, 1.060) | 0.982 |
| Global | Lag 0–2 | 1.038 (1.002, 1.075) | 0.041 |
| Asia | Lag 0 | 1.071 (0.989, 1.159) | 0.091 |
| Asia | Lag 1 | 1.049 (0.970, 1.134) | 0.234 |
| Asia | Lag 2 | 1.049 (0.971, 1.133) | 0.226 |
| Asia | Lag 0–2 | 1.059 (1.010, 1.111) | 0.017 |
| Africa | Lag 0 | 1.213 (1.090, 1.349) | <0.001 |
| Africa | Lag 1 | 1.008 (0.900, 1.128) | 0.894 |
| Africa | Lag 2 | 1.010 (0.905, 1.126) | 0.862 |
| Africa | Lag 0–2 | 1.073 (1.002, 1.149) | 0.045 |
| Latin America | Lag 0 | 1.017 (0.870, 1.188) | 0.836 |
| Latin America | Lag 1 | 0.882 (0.752, 1.035) | 0.125 |
| Latin America | Lag 2 | 0.837 (0.685 ,1.024) | 0.376 |
| Latin America | Lag 0–2 | 0.909 (0.822, 1.005) | 0.063 |
