# Supplementary material for: Cyclone exposure and mortality risk of children under 5 years old: An observational study in 34 low- and middle-income countries
Source: PLoS Med. 2025 Sep 25;22(9):e1004735. doi: 10.1371/journal.pmed.1004735 (PMC12463208; doi:10.1371/journal.pmed.1004735)
Supplement: S3 Table — (DOCX) [file pmed.1004735.s005.docx]

**S3 Table. Odds ratios (95% confidence intervals) of death risks in children under 5 years old associated with exposure of cyclone in the first month before death in models of stratification analyses.**

| Characteristics | Odds ratio (95% CI) | Z value | P value |
| --- | --- | --- | --- |
| Residence area |  |  |  |
| Urban | 1.154 (1.026, 1.299) | Ref |  |
| Rural | 1.087 (1.017, 1.161) | 0.875 | 0.382 |
| Children’s gender |  |  |  |
| Male | 1.114 (1.020, 1.217) | Ref |  |
| Female | 1.093 (0.992, 1.204) | 0.286 | 0.775 |
| Birth order |  |  |  |
| First child | 1.059 (0.985, 1.138) | Ref |  |
| Not first child | 1.208 (0.906, 1.610) | 0.870 | 0.384 |
| Mother’s highest education |  |  |  |
| Primary or no education | 1.101 (1.032, 1.174) | Ref |  |
| Secondary education | 1.101 (0.960, 1.263) | 0.005 | 0.996 |
| High school or above | 1.059 (0.719, 1.560) | 0.192 | 0.847 |
| Regional GDP per capita |  |  |  |
| Lower than average | 1.190 (1.083, 1.307) | Ref |  |
| Higher than average | 1.057 (0.982, 1.138) | 1.936 | 0.053 |
| Regional medical resource |  |  |  |
| Lower than average | 1.164 (1.069, 1.268) | Ref |  |
| Higher than average | 1.061 (0.979, 1.149) | 1.565 | 0.117 |
| Water sources |  |  |  |
| Piped or bottled water | 1.041 (0.928, 1.167) | Ref |  |
| Well water | 1.105 (1.014, 1.205) | 0.826 | 0.409 |
| Natural water | 1.216 (1.061, 1.395) | 1.713 | 0.087 |
| Others | 1.117 (0.923, 1.352) | 0.623 | 0.533 |
| Toilet types |  |  |  |
| Flush toilet | 1.014 (0.895, 1.149) | Ref |  |
| Pit toilet | 1.146 (1.047, 1.255) | 1.559 | 0.119 |
| No toilet | 1.113 (1.003, 1.236) | 1.128 | 0.259 |
| Others | 1.086 (0.840, 1.403) | 0.468 | 0.640 |
| Household materials |  |  |  |
| Unfinished | 1.155 (1.069, 1.248) | Ref |  |
| Finished | 1.042 (0.955, 1.138) | 1.720 | 0.085 |
