# Supplementary material for: Cyclone exposure and mortality risk of children under 5 years old: An observational study in 34 low- and middle-income countries
Source: PLoS Med. 2025 Sep 25;22(9):e1004735. doi: 10.1371/journal.pmed.1004735 (PMC12463208; doi:10.1371/journal.pmed.1004735)
Supplement: S4 Table — (DOCX) [file pmed.1004735.s006.docx]

**S4 Table. Odds ratios (95% confidence intervals) of death risks in children under 5 years old associated with exposure of cyclone in models of stratification analyses.**

| Characteristics | Lag 0 month | Lag 1 month | Lag 2 month | Lag 0–2 months |
| --- | --- | --- | --- | --- |
| Residence area |  |  |  |  |
| Rural | 1.087 (1.017, 1.161) | 1.018 (0.953, 1.088) | 1.023 (0.959, 1.093) | 1.027 (0.953 ,1.108) |
| Urban | 1.154 (1.026, 1.299) | 0.978 (0.861, 1.111) | 0.928 (0.818, 1.053) | 1.042 (1.001 ,1.085) |
| Children’s gender |  |  |  |  |
| Male | 1.114 (1.020, 1.217) | 1.072 (0.981, 1.170) | 1.002 (0.918, 1.094) | 1.062 (1.007 ,1.121) |
| Female | 1.093 (0.992, 1.204) | 0.963 (0.871, 1.064) | 1.027 (0.932, 1.133) | 1.031 (0.971 ,1.095) |
| Birth order |  |  |  |  |
| First child | 1.059 (0.985, 1.138) | 0.986 (0.916, 1.061) | 1.029 (0.958, 1.105) | 1.048 (0.876 ,1.254) |
| Not first child | 1.208 (0.906, 1.610) | 0.939 (0.704, 1.252) | 0.981 (0.719, 1.340) | 1.029 (0.985 ,1.076) |
| Mother’s highest education |  |  |  |  |
| Primary or no education | 1.101 (1.032, 1.174) | 0.998 (0.935, 1.066) | 1.022 (0.959, 1.090) | 1.042 (1.002 ,1.084) |
| Secondary education | 1.101 (0.960, 1.263) | 1.067 (0.929, 1.226) | 0.901 (0.782, 1.039) | 1.014 (0.932 ,1.104) |
| High school or above | 1.059 (0.719, 1.560) | 0.939 (0.623, 1.415) | 1.019 (0.686, 1.515) | 1.027 (0.794 ,1.327) |
| Regional GDP per capita |  |  |  |  |
| Lower than average | 1.190 (1.083, 1.307) | 1.035 (0.940, 1.140) | 1.076 (0.980, 1.180) | 1.105 (1.042 ,1.171) |
| Higher than average | 1.057 (0.982, 1.138) | 0.999 (0.928, 1.077) | 0.960 (0.891, 1.035) | 1.005 (0.960 ,1.051) |
| Regional medical resource |  |  |  |  |
| Lower than average | 1.164 (1.069, 1.268) | 1.077 (0.988, 1.175) | 1.072 (0.984, 1.167) | 1.110 (1.053, 1.170) |
| Higher than average | 1.061 (0.979, 1.149) | 0.970 (0.895, 1.051) | 0.954 (0.880, 1.034) | 0.993 (0.946, 1.043) |
| Water sources |  |  |  |  |
| Piped or bottled water | 1.041 (0.928, 1.167) | 1.022 (0.912, 1.144) | 0.978 (0.873, 1.095) | 1.007 (0.939 ,1.079) |
| Well water | 1.105 (1.014, 1.205) | 1.029 (0.944, 1.121) | 1.042 (0.957, 1.134) | 1.064 (1.009 ,1.121) |
| Natural water | 1.216 (1.061, 1.395) | 1.052 (0.911, 1.215) | 0.960 (0.835, 1.104) | 1.083 (0.993 ,1.180) |
| Others | 1.117 (0.923, 1.352) | 0.847 (0.692, 1.036) | 0.916 (0.748, 1.121) | 0.949 (0.842 ,1.071) |
| Toilet types |  |  |  |  |
| Flush toilet | 1.014 (0.895, 1.149) | 1.052 (0.932, 1.187) | 1.011 (0.893, 1.144) | 1.011 (0.937 ,1.090) |
| Pit toilet | 1.146 (1.047, 1.255) | 1.042 (0.949, 1.144) | 1.035 (0.944, 1.134) | 1.085 (1.026 ,1.147) |
| No toilet | 1.113 (1.003, 1.236) | 0.983 (0.885, 1.092) | 0.964 (0.869, 1.070) | 1.014 (0.952 ,1.081) |
| Others | 1.086 (0.840, 1.403) | 0.864 (0.656, 1.139) | 1.035 (0.810, 1.322) | 1.022 (0.873 ,1.197) |
| Household materials |  |  |  |  |
| Unfinished | 1.155 (1.069, 1.248) | 0.978 (0.903, 1.059) | 1.037 (0.961, 1.119) | 1.057 (1.008 ,1.108) |
| Finished | 1.042 (0.955, 1.138) | 1.061 (0.972, 1.158) | 0.962 (0.879, 1.052) | 1.022 (0.969 ,1.079) |
