# Supplementary material for: Cyclone exposure and mortality risk of children under 5 years old: An observational study in 34 low- and middle-income countries
Source: PLoS Med. 2025 Sep 25;22(9):e1004735. doi: 10.1371/journal.pmed.1004735 (PMC12463208; doi:10.1371/journal.pmed.1004735)
Supplement: S5 Table — (DOCX) [file pmed.1004735.s007.docx]

**S5 Table. Odds ratio (95% confidence intervals) of death risks in children under 5 years old associated with exposure of cyclone in the first month before death in models of sensitivity analyses**

| Model | Lag (months) | Odds ratio (95% CI) | P value |
| --- | --- | --- | --- |
| Main model | Lag 0 | 1.101 (1.039, 1.166) | 0.001 |
|  | Lag 1 | 1.009 (0.952, 1.070) | 0.754 |
|  | Lag 2 | 1.001 (0.945, 1.060) | 0.982 |
|  | Lag 0–2 | 1.038 (1.002 ,1.075) | 0.041 |
| Model 1 | Lag 0 | 1.098 (1.037, 1.163) | 0.001 |
|  | Lag 1 | 1.010 (0.953, 1.070) | 0.747 |
|  | Lag 2 | 1.000 (0.944, 1.059) | 0.996 |
|  | Lag 0–2 | 1.036 (1.000 ,1.074) | 0.047 |
| Model 2 | Lag 0 | 1.100 (1.039, 1.165) | 0.001 |
|  | Lag 1 | 1.009 (0.952, 1.070) | 0.760 |
|  | Lag 2 | 1.001 (0.945, 1.061) | 0.962 |
|  | Lag 0–2 | 1.038 (1.002 ,1.075) | 0.039 |
| Model 3 | Lag 0 | 1.102 (1.040, 1.168) | 0.001 |
|  | Lag 1 | 0.995 (0.938, 1.055) | 0.869 |
|  | Lag 2 | 1.000 (0.943, 1.060) | 0.998 |
|  | Lag 0–2 | 1.035 (0.998 ,1.073) | 0.061 |

Notes: The covariates of *Main model* included the death month of cases, monthly cumulative temperature and monthly cumulative precipitation. *Model 1* changed the degree of freedom of temperature and precipitation from 3 to 6. The covariates of *Model 2* included the covariates in the *Main model* and sex of children. The covariates of *Model 3* included the covariates in the *Main model* except the death year and month of cases.
