# Supplementary material for: Cyclone exposure and mortality risk of children under 5 years old: An observational study in 34 low- and middle-income countries
Source: PLoS Med. 2025 Sep 25;22(9):e1004735. doi: 10.1371/journal.pmed.1004735 (PMC12463208; doi:10.1371/journal.pmed.1004735)
Supplement: S6 Table — (DOCX) [file pmed.1004735.s008.docx]

**S6 Table. Odds ratio (95% confidence intervals) of death risks in children under 5 years old associated with exposure of cyclone in the first month before death after excluding every single country.**

| Excluded country | Excluded case number | Excluded control number | Odds ratio (%) |
| --- | --- | --- | --- |
| Null | 0 | 0 | 1.101 (1.039, 1.166) |
| Bangladesh | 14,612 | 37,877 | 1.103 (1.034, 1.178) |
| Benin | 69 | 232 | 1.099 (1.038, 1.164) |
| Burkina Faso | 760 | 1,947 | 1.098 (1.036, 1.163) |
| Cambodia | 3,380 | 8,420 | 1.103 (1.040, 1.169) |
| Cameroon | 44 | 106 | 1.101 (1.039, 1.166) |
| Colombia | 364 | 1,030 | 1.100 (1.038, 1.165) |
| Dominican Republic | 2,856 | 8,182 | 1.103 (1.040, 1.170) |
| Eswatini | 71 | 178 | 1.101 (1.039, 1.166) |
| Ethiopia | 1,372 | 4,013 | 1.100 (1.038, 1.165) |
| Ghana | 124 | 374 | 1.099 (1.038, 1.164) |
| Guatemala | 1,759 | 6,040 | 1.100 (1.039, 1.165) |
| Guinea | 604 | 1,493 | 1.100 (1.038, 1.165) |
| Haiti | 6,083 | 16,858 | 1.108 (1.043, 1.177) |
| Honduras | 1,624 | 5,731 | 1.099 (1.038, 1.165) |
| India | 45,668 | 98,123 | 1.141 (1.070, 1.218) |
| Indonesia | 103 | 297 | 1.101 (1.039, 1.166) |
| Kenya | 176 | 546 | 1.098 (1.036, 1.163) |
| Madagascar | 7,852 | 21,621 | 1.095 (1.029, 1.165) |
| Malawi | 723 | 1,982 | 1.100 (1.038, 1.165) |
| Mali | 325 | 874 | 1.098 (1.036, 1.163) |
| Mozambique | 1,658 | 3,496 | 1.102 (1.040, 1.167) |
| Myanmar | 1,724 | 3,981 | 1.104 (1.042, 1.170) |
| Namibia | 72 | 198 | 1.100 (1.039, 1.165) |
| Nigeria | 3,044 | 6,699 | 1.103 (1.040, 1.170) |
| Pakistan | 588 | 1,690 | 1.100 (1.038, 1.165) |
| Philippines | 1,968 | 6,560 | 1.090 (1.027, 1.157) |
| Senegal | 214 | 752 | 1.099 (1.038, 1.164) |
| Sierra Leone | 329 | 690 | 1.100 (1.039, 1.165) |
| Timor Leste | 668 | 2,045 | 1.100 (1.039, 1.165) |
| Togo | 178 | 505 | 1.101 (1.039, 1.166) |
| Uganda | 254 | 848 | 1.099 (1.038, 1.164) |
| United Republic of Tanzania | 606 | 1,670 | 1.097 (1.035, 1.162) |
| Zambia | 175 | 552 | 1.097 (1.035, 1.162) |
| Zimbabwe | 751 | 1,835 | 1.100 (1.038, 1.165) |
