# Supplementary material for: Cyclone exposure and mortality risk of children under 5 years old: An observational study in 34 low- and middle-income countries
Source: PLoS Med. 2025 Sep 25;22(9):e1004735. doi: 10.1371/journal.pmed.1004735 (PMC12463208; doi:10.1371/journal.pmed.1004735)
Supplement: S7 Table — (DOCX) [file pmed.1004735.s009.docx]

**S7 Table. Odds ratio (95% confidence intervals) of death risks in children under 1 years old associated with exposure of cyclone**

| Lag (months) | Odds ratio (95% CI) | P value |
| --- | --- | --- |
| Lag 0 | 1.109 (1.036, 1.186) | 0.003 |
| Lag 1 | 0.994 (0.928, 1.064) | 0.866 |
| Lag 2 | 1.016 (0.950, 1.086) | 0.645 |
| Lag 0–2 | 1.040 (0.998 ,1.084) | 0.063 |
