# Supplementary material for: Cyclone exposure and mortality risk of children under 5 years old: An observational study in 34 low- and middle-income countries
Source: PLoS Med. 2025 Sep 25;22(9):e1004735. doi: 10.1371/journal.pmed.1004735 (PMC12463208; doi:10.1371/journal.pmed.1004735)
Supplement: S8 Table — (DOCX) [file pmed.1004735.s010.docx]

**S8 Table. Odds ratios (and 95% confidence intervals) of diarrhea risks in children under 5 years old associated with cyclone exposure, stratified by water sources and toilet types.**

| Groups | Sample size | Case number | Lag periods  (month) | Odds ratio (95%CI) | P value |
| --- | --- | --- | --- | --- | --- |
| Global | 688,836 | 97,450 | 0 | 1.280 (1.114,1.472) | 0.001 |
|  |  |  | 0–2 | 1.224 (1.144,1.309) | <0.001 |
| Water sources |  |  |  |  |  |
| Piped or bottled water | 39,731 | 5,527 | 0 | 0.884 (0.432, 1.807) | 0.735 |
|  |  |  | 0–2 | 1.200 (1.020, 1.412) | 0.028 |
| Well water | 492,908 | 70,450 | 0 | 1.239 (1.029, 1.491) | 0.024 |
|  |  |  | 0–2 | 1.284 (1.173, 1.406) | <0.001 |
| Natural water | 112,787 | 16,095 | 0 | 1.669 (1.333, 2.089) | <0.001 |
|  |  |  | 0–2 | 1.444 (1.254, 1.664) | <0.001 |
| Toilet type |  |  |  |  |  |
| Flush toilet | 141,001 | 17,615 | 0 | 1.035 (0.798, 1.344) | 0.794 |
|  |  |  | 0–2 | 1.133 (1.010, 1.271) | 0.033 |
| Pit toilet | 339,307 | 50,977 | 0 | 1.104 (0.821, 1.483) | 0.514 |
|  |  |  | 0–2 | 1.253 (1.104, 1.422) | <0.001 |
| No toilet | 186,937 | 26,787 | 0 | 1.469 (1.199, 1.799) | <0.001 |
|  |  |  | 0–2 | 1.221 (1.081, 1.379) | <0.001 |
