# Supplementary material for: Cyclone exposure and mortality risk of children under 5 years old: An observational study in 34 low- and middle-income countries
Source: PLoS Med. 2025 Sep 25;22(9):e1004735. doi: 10.1371/journal.pmed.1004735 (PMC12463208; doi:10.1371/journal.pmed.1004735)
Supplement: S9 Table — (DOCX) [file pmed.1004735.s011.docx]

**S9 Table. Comparison of annual estimated population and deaths of children under 5 in DHS countries (2000–2020) with UNICEF and WorldBank database**

| Country | Estimated population under 5 years old (10,000) | Population under 5 years old from WorldBank (10,000) | Estimated total deaths under 5 years old (10,000) | Total deaths under 5 years old from UNICEF database (10,000 with uncertainty bounds) |
| --- | --- | --- | --- | --- |
| Bangladesh | 1,514.89 | 2,022.49 | 16.66 | 18.76  (18.08, 19.46) |
| Benin | 158 | 173.62 | 3.73 | 4.03  (3.68, 4.41) |
| Burkina Faso | 293.15 | 299.03 | 8.05 | 8.01  (7.02, 9.2) |
| Cambodia | 178.02 | 204.76 | 1.86 | 1.84  (1.52, 2.29) |
| Cameroon | 364.28 | 336.4 | 8.59 | 7.94  (7.18, 8.78) |
| Colombia | 428.37 | 468.02 | 1.57 | 1.46  (1.27, 1.69) |
| Dominican Republic | 98.08 | 122.93 | 0.73 | 0.76  (0.67, 0.87) |
| Eswatini | 18.93 | 18.48 | 0.3 | 0.25  (0.22, 0.29) |
| Ethiopia | 1,382.18 | 1809.08 | 25.67 | 28.7  (26.25, 31.44) |
| Ghana | 327.25 | 373.52 | 4.7 | 5.31  (4.94, 5.71) |
| Guatemala | 202.77 | 233.79 | 1.5 | 1.45  (1.28, 1.64) |
| Guinea | 166.29 | 176.91 | 4.67 | 4.96  (4.52, 5.46) |
| Haiti | 124.69 | 147.04 | 2.2 | 2.19  (1.92, 2.51) |
| Honduras | 99.93 | 129.32 | 0.53 | 0.57  (0.5, 0.64) |
| India | 12,254.94 | 15,403.56 | 151.93 | 161.7  (154.31, 169.59) |
| Indonesia | 2,221.17 | 2,811.22 | 15.94 | 16.91  (15.58, 18.44) |
| Kenya | 639.3 | 655.24 | 8 | 8.31  (7.66, 9.02) |
| Madagascar | 333.72 | 431.99 | 5.44 | 5.94  (5.4, 6.54) |
| Malawi | 241.13 | 260.13 | 4.54 | 4.92  (4.41, 5.5) |
| Mali | 299.31 | 307.02 | 9.04 | 9.33  (8.5, 10.27) |
| Mozambique | 394.82 | 491.5 | 8.72 | 9.19  (8.4, 10.04) |
| Myanmar | 536.2 | 554.13 | 7.75 | 6.47  (5.54, 7.51) |
| Namibia | 27.55 | 30.08 | 0.35 | 0.38  (0.32, 0.44) |
| Nigeria | 2,723.55 | 2,909.61 | 85.07 | 91.11  (81.43, 102.97) |
| Pakistan | 2,403.24 | 3,374.25 | 44.68 | 53.02  (49.65, 56.71) |
| Philippines | 1,124.81 | 1,399.36 | 7.02 | 7.29  (6.62, 8.05) |
| Senegal | 219.41 | 207.88 | 3.31 | 3.17  (2.96, 3.4) |
| Sierra Leone | 94.51 | 100.19 | 3.5 | 3.67  (3.38, 3.99) |
| Timor Leste | 100.38 | 108.01 | 1.92 | 2.06  (1.85, 2.31) |
| Togo | 629.22 | 612.8 | 10.94 | 10.74  (9.71, 11.93) |
| Uganda | 751.04 | 789.05 | 11.98 | 12.45  (11.44, 13.57) |
| United Republic of Tanzania | 246.89 | 249.4 | 4.42 | 4.52  (4.15, 4.91) |
| Zambia | 186.34 | 254.8 | 3.08 | 3.54  (3.14, 3.98) |
| Zimbabwe | 1,514.89 | 2,022.49 | 16.66 | 18.76  (18.08, 19.46) |
| Total annual average | 30,784.36 | 37,465.61 | 468.39 | 500.94  (463.50, 543.56) |
| Total | 646,471.55 | 786,777.74 | 9,836.17 | 10,519.75  (9,733.52, 11,414.73) |
