# Supplementary material for: Cyclone exposure and mortality risk of children under 5 years old: An observational study in 34 low- and middle-income countries
Source: PLoS Med. 2025 Sep 25;22(9):e1004735. doi: 10.1371/journal.pmed.1004735 (PMC12463208; doi:10.1371/journal.pmed.1004735)
Supplement: S10 Table — (DOCX) [file pmed.1004735.s012.docx]

**S10 Table. Total deaths in children under 5 years old attributable to cyclone exposures in the first month before death in DHS countries exposed to cyclones from 2000 to 2020.**

| Region | Country | Attributable deaths under 5 years old (1,000 and 95% CI) | Attributable excess mortality rate under 5 years old (1/10,000 and 95% CI) |
| --- | --- | --- | --- |
| Africa |  | 157.5  (64.45, 244.43) | 2.46  (1.01, 3.82) |
|  | Ethiopia | 0.68  (0.28, 1.05) | 0.02  (0.01, 0.04) |
|  | Madagascar | 90.09  (36.86, 139.82) | 12.86  (5.26, 19.95) |
|  | Mozambique | 62.58  (25.61, 97.12) | 7.55  (3.09, 11.71) |
|  | United Republic of Tanzania | 0.22  (0.09, 0.35) | 0.01  (0.01, 0.02) |
|  | Zimbabwe | 3.93  (1.61, 6.1) | 1.01  (0.41, 1.56) |
| Asia |  | 630.43  (257.96, 978.39) | 0.79  (0.32, 1.23) |
|  | Bangladesh | 70.48  (28.84, 109.38) | 2.22  (0.91, 3.44) |
|  | Cambodia | 0.5  (0.2, 0.77) | 0.13  (0.05, 0.21) |
|  | East Timor | 0.003  (0.001, 0.004) | 0.007  (0.003, 0.011) |
|  | India | 207.94  (85.09, 322.71) | 0.81  (0.33, 1.25) |
|  | Indonesia | 0.64  (0.26, 1) | 0.014  (0.006, 0.022) |
|  | Myanmar | 33.6  (13.75, 52.15) | 2.98  (1.22, 4.63) |
|  | Pakistan | 4.59  (1.88, 7.13) | 0.09  (0.04, 0.14) |
|  | Philippines | 312.67  (127.94, 485.24) | 13.24  (5.42, 20.54) |
| Latin America |  | 65.05  (26.62, 100.95) | 3.25  (1.33, 5.04) |
|  | Colombia | 0.12  (0.05, 0.19) | 0.01  (0.01, 0.02) |
|  | Dominican Republic | 14.28  (5.84, 22.16) | 6.93  (2.84, 10.76) |
|  | Guatemala | 2.21  (0.9, 3.42) | 0.52  (0.21, 0.8) |
|  | Haiti | 46.15  (18.88, 71.62) | 17.63  (7.21, 27.36) |
|  | Honduras | 2.3  (0.94, 3.56) | 1.09  (0.45, 1.7) |
| Total |  | 852.98  (349.02, 1323.77) | 1.67  (0.69, 2.6) |
