# Supplementary material for: Cyclone exposure and mortality risk of children under 5 years old: An observational study in 34 low- and middle-income countries
Source: PLoS Med. 2025 Sep 25;22(9):e1004735. doi: 10.1371/journal.pmed.1004735 (PMC12463208; doi:10.1371/journal.pmed.1004735)
Supplement: S1 STROBE Checklist — This checklist is licensed under the Creative Commons Attribution 4.0 International License (CC BY 4.0; https://creativecommons.org/licenses/by/4.0/). (DOCX) [file pmed.1004735.s013.docx]

STROBE Statement—Checklist of items that should be included in reports of ***case-control studies***

|  | **Item No** | **Recommendation** | **Section and paragraph numbers** |
| --- | --- | --- | --- |
| **Title and abstract** | 1 | (*a*) Indicate the study’s design with a commonly used term in the title or the abstract | Title and Abstract paragraph (para.) 2 |
|  |  | (*b*) Provide in the abstract an informative and balanced summary of what was done and what was found | Abstract para. 2 |
| **Introduction** | | | |
| Background/rationale | 2 | Explain the scientific background and rationale for the investigation being reported | Introduction para. 1-3 |
| Objectives | 3 | State specific objectives, including any prespecified hypotheses | Introduction para. 3 |
| **Methods** | | | |
| Study design | 4 | Present key elements of study design early in the paper | Introduction para. 3 |
| Setting | 5 | Describe the setting, locations, and relevant dates, including periods of recruitment, exposure, follow-up, and data collection | Method and Materials para. 1,3,5,6 |
| Participants | 6 | (*a*) Give the eligibility criteria, and the sources and methods of case ascertainment and control selection. Give the rationale for the choice of cases and controls | Method and Materials para. 2 |
|  |  | (*b*) For matched studies, give matching criteria and the number of controls per case | Method and Materials para. 3 |
| Variables | 7 | Clearly define all outcomes, exposures, predictors, potential confounders, and effect modifiers. Give diagnostic criteria, if applicable | Method and Materials para. 2,3,7,9,10 |
| Data sources/ measurement | 8* | For each variable of interest, give sources of data and details of methods of assessment (measurement). Describe comparability of assessment methods if there is more than one group | Method and Materials para. 1,3-8 |
| Bias | 9 | Describe any efforts to address potential sources of bias | Method and Materials para. 8-12 |
| Study size | 10 | Explain how the study size was arrived at | Results para. 1 |
| Quantitative variables | 11 | Explain how quantitative variables were handled in the analyses. If applicable, describe which groupings were chosen and why | Method and Materials para. 8,9 |
| Statistical methods | 12 | (*a*) Describe all statistical methods, including those used to control for confounding | Method and Materials para. 8-10 |
|  |  | (*b*) Describe any methods used to examine subgroups and interactions | Method and Materials para. 10,11 |
|  |  | (*c*) Explain how missing data were addressed | Results para. 1 |
|  |  | (*d*) If applicable, explain how matching of cases and controls was addressed | Method and Materials para. 3 |
|  |  | (*e*) Describe any sensitivity analyses | Method and Materials para. 13 |
| **Results** | | | |
| Participants | 13* | (a) Report numbers of individuals at each stage of study—eg numbers potentially eligible, examined for eligibility, confirmed eligible, included in the study, completing follow-up, and analysed | Results para. 1 |
|  |  | (b) Give reasons for non-participation at each stage | Results para. 1 |
|  |  | (c) Consider use of a flow diagram | Figure 1 |
| Descriptive data | 14* | (a) Give characteristics of study participants (eg demographic, clinical, social) and information on exposures and potential confounders | Results para. 1; Table 1 and sTable 1 |
|  |  | (b) Indicate number of participants with missing data for each variable of interest | Results para. 1 |
| Outcome data | 15* | Report numbers in each exposure category, or summary measures of exposure | Results para. 1; Table 1 |

| Main results | | 16 | (*a*) Give unadjusted estimates and, if applicable, confounder-adjusted estimates and their precision (eg, 95% confidence interval). Make clear which confounders were adjusted for and why they were included | Results para. 5; sTable 5 and 6 |
| --- | --- | --- | --- | --- |
|  |  |  | (*b*) Report category boundaries when continuous variables were categorized | N/A |
|  |  |  | (*c*) If relevant, consider translating estimates of relative risk into absolute risk for a meaningful time period | N/A |
| Other analyses | 17 | Report other analyses done—eg analyses of subgroups and interactions, and sensitivity analyses | | Results para. 4,6,7; Figure 4, sTbale 2, sTbale 3, sTbale 7-10, |
| **Discussion** | | | |  |
| Key results | 18 | Summarise key results with reference to study objectives | | Disscusion para. 1 |
| Limitations | 19 | Discuss limitations of the study, taking into account sources of potential bias or imprecision. Discuss both direction and magnitude of any potential bias | | Disscusion para. 6 |
| Interpretation | 20 | Give a cautious overall interpretation of results considering objectives, limitations, multiplicity of analyses, results from similar studies, and other relevant evidence | | Disscusion para. 7 |
| Generalisability | 21 | Discuss the generalisability (external validity) of the study results | | Disscusion para. 6 |
| **Other information** | | | |  |
| Funding | 22 | Give the source of funding and the role of the funders for the present study and, if applicable, for the original study on which the present article is based | | Financial Disclosure statement |

*Give information separately for cases and controls.

**Note:** An Explanation and Elaboration article discusses each checklist item and gives methodological background and published examples of transparent reporting. The STROBE checklist is best used in conjunction with this article (freely available on the Web sites of PLoS Medicine at http://www.plosmedicine.org/, Annals of Internal Medicine at http://www.annals.org/, and Epidemiology at http://www.epidem.com/). Information on the STROBE Initiative is available at http://www.strobe-statement.org.
